# Co-crystal of nadifloxacin with oxalic acid

**DOI:** 10.1107/S2056989023002244

**Published:** 2023-03-10

**Authors:** Geethanjali N. Karthammaiah, Sreenivasa Rao Amaraneni, Anand K. Solomon

**Affiliations:** a Dayananda Sagar University, Karnataka, India; Tulane University, USA

**Keywords:** crystal structure, slow evaporation, hydrogen bonding, Hirshfeld surface analysis, anti­bacterial drug

## Abstract

The 2:1 co-crystal of nadifloxacin [systematic name: 9-fluoro-8-(4-hy­droxy­piperidin-1-yl)-5-methyl-1-oxo-6,7-di­hydro-1*H*,5*H*-pyrido[3,2,1-*ij*]quinoline-2-carb­oxy­lic acid] with oxalic acid, C_19_H_21_FN_2_O_4_·0.5C_2_H_2_O_4_, was prepared by slow evaporation from a chloro­form:acetone solvent system. Nadifloxacin belongs to the group of anti­bacterial drugs. The co-crystal is stabilized through an intra­molecular O—H⋯O bond and inter­molecular hydrogen bonds. It was studied by FT–IR spectroscopy, differential scanning calorimetry and X-ray diffraction. Hirshfeld surface analysis indicated that the major contribution to the packing is from O⋯H/H⋯O inter­actions.

## Abstract 2

The nadifloxacin oxalic acid co-crystal is stabilized by inter­molecular hydrogen bonds. FT–IR, DSC and XRD studies were carried out to confirm the structure and a Hirshfeld surface analysis was performed to investigate the inter­molecular inter­actions.

## 1. Chemical context

A co-crystal is a multi-component mol­ecular complex with a definite stoichiometric ratio of two compounds that can inter­act through hydrogen bonds, van der Waals forces, and π-stacking inter­actions to name just a few (Stahly 2009 ▸; Vishweshwar *et al.*, 2006 ▸). The formation of multi-component crystals, *i.e.* salts and co-crystals through a crystal-engineering approach has been demonstrated to be a versatile tool to improve the physicochemical properties of APIs (active pharmaceutical ingredients) including solubility, dissolution rate, stability, tabletability, *etc*. (Mannava *et al.*, 2021 ▸, 2022 ▸). Co-crystals can be synthesized by various methods such as solvent-assisted grinding, sonication and slow evaporation among others. Co-crystals of fluoro­quinolone anti­biotics with organic acids have been reported to exhibit higher solubility than the parent mol­ecule (Reddy *et al.*, 2011 ▸). Nadifloxacin fluoro­quinolone (Kido & Hashimoto, 1994 ▸) is an anti­biotic used for the treatment of commonly formed acne, acting against *Staphylococcus aureus*, *Streptococcus* spp., coagulase-negative *staphylococci* (CNS), *Propionibacterium acnes*, and *Propionibacterium granulosum* strains (Nenoff *et al.*, 2004 ▸). It also shows anti­bacterial activity against skin infections (Kumar & Khatak, 2021 ▸). Here we report the structure of a co-crystal formed between nadifloxacin (NAD) and oxalic acid (OA), which is stabilized through inter­molecular hydrogen bonds. 
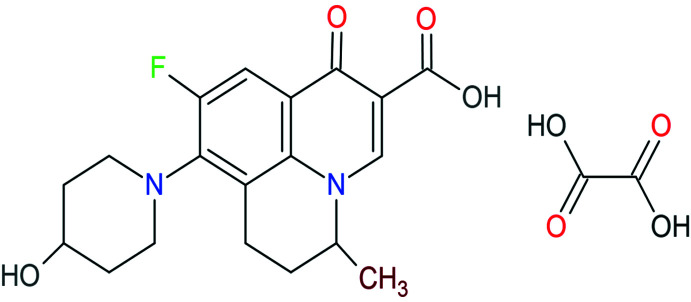


## 2. Structural commentary

**Figure 1:**
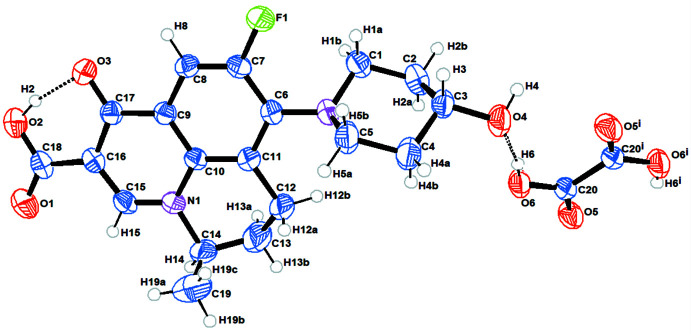
A perspective view of the title compound with labeling scheme and 50% probability ellipsoids. Symmetry code: (i) −*x* + 2, −*y* + 2, −*z* + 2.

The title co-crystal is shown in Fig. 1 ▸. The asymmetric unit is comprised of one NAD mol­ecule in a general position and half of an OA mol­ecule, located about a center of inversion, so the co-crystal is formulated as a 2:1 NAD:OA adduct. NAD is a non-planar mol­ecule [C7—C6—N2—C5 = 104.0 (3)°]. The adduct forms through O6—H6⋯O4 hydrogen bonds (Table 1 ▸) and crystallizes in the triclinic crystal system in space group *P* 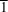 . An intra­molecular O2—H2⋯O3 hydrogen bond is formed in the NAD mol­ecule with an 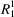 (6) ring motif of (Fig. 1 ▸).

## 3. Supra­molecular features

**Figure 2:**
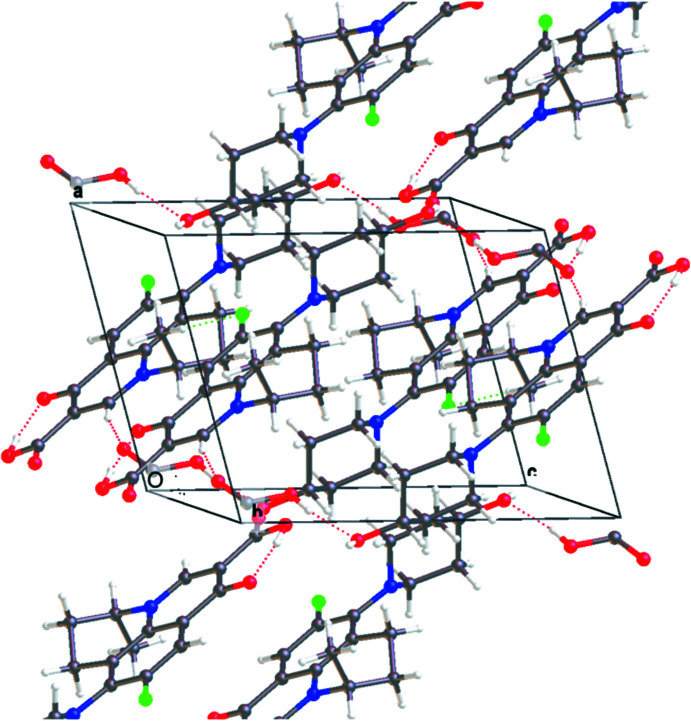
Packing of the title compound with hydrogen bonds depicted by dashed lines.

**Figure 3:**
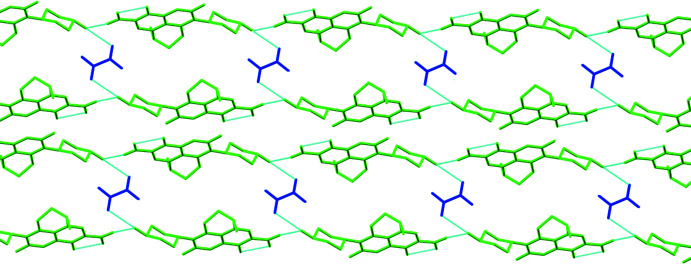
Two ribbons of NAD and OA formed by hydrogen bonds.

**Figure 4:**
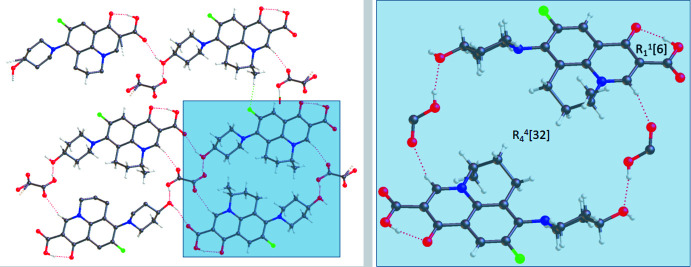
The ring motif between oxalic acid and nadifloxacin in the co-crystal.

In the crystal, O4—H4⋯O1^iii^ hydrogen bonds (Fig. 2 ▸, Table 1 ▸) forms chains of NAD mol­ecules [graph-set motif *S* (6) (Etter *et al.*, 1990 ▸)] extending parallel to (10 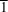 ). The chains are linked by inversion-related O6—H6⋯O4 hydrogen bonds between NAD and OA, forming ribbons of the 2:1 adducts parallel to (11 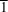 ) (Fig. 3 ▸). The hydroxyl oxygen O4 of NAD is involved in a bifurcated inter­action acting as both acceptor (O6—H6⋯O4) and donor (O4—H4⋯O1^iii^). A larger ring motif is formed with two mol­ecules of nadifloxacin and two mol­ecules of oxalic acid having an 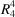 (32) graph-set motif (Fig. 4 ▸).

## 4. Database survey

A number of related structures have been reported in the literature, including a norfloxacin–oxalate dihydrate adduct with an 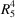 (12) ring motif and ciprofloxacin malonate dihydrate in which an R_4_^4^(16) ring motif is observed. Both adducts are connected through tetra­meric clusters of water mol­ecules (Reddy *et al.*, 2011 ▸). In the ofloxacin adduct with diphenic acid, the components are connected by charge-assisted strong bifurcated N—H^+^⋯O hydrogen bonds, forming 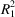 (4) ring motifs (Suresh *et al.*, 2020 ▸). An enrofloxacin–pimelic acid adduct shows an 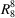 (20) ring motif of (Yang *et al.*, 2022 ▸), a norfloxacin-pimelic acid adduct an 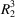 (8) ring motif while in the structure of a ciprofloxacin–suberic acid co-crystal, 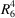 (12) and 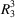 (15) ring motifs are observed (O’Malley *et al.*, 2022 ▸) and a pefloxacin–oxalic acid salt forms an 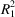 (5) ring motif (Nangia *et al.*, 2018 ▸). Several polymorphs of nalidixic acid and co-crystals of it with various hydroxyl compounds show bifurcated hydrogen bonds (Gangavaram *et al.*, 2012 ▸), while a nicorandil–fumaric acid co-crystal features hydrogen bonds with a dimeric 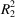 (1) ring motif (Mannava *et al.*, 2021 ▸, 2022 ▸). Pharmaceuticals co-crystals (Vishweshwar *et al.*, 2006 ▸) pave way for new chemical entities with tuned physicochemical properties.

## 5. Synthesis and crystallization

NAD was purchased from Swapnroop Drugs and Pharmaceuticals, India, and the remaining chemicals were purchased from Sigma-Aldrich, India. All chemicals and solvents were of analytical grade.

NAD (50 mg, 0.360 mmol) and OA (17 mg, 0.126 mmol) were dissolved in a mixed chloro­form–acetone solvent (5 ml:5 ml), heated on a water bath for 15–20 min and then kept undisturbed for slow evaporation. Crystals were obtained at room temperature after 24-48 h. They were characterized by FTIR, DSC, and single crystal XRD.

Infrared spectra of NAD·OA crystals were recorded using FT–IR spectroscopy (Thermo-Nicolet 6700 FTIR–NIR spectrometer) with the samples made in KBr pellets. *Omnic* software (Thermo Scientific, Waltham, MA) was used to analyze the data. Each sample was scanned in the range 400-4000 cm^−1^

In the IR spectrum, the C=O stretching frequencies for NAD (carb­oxy­lic acid group) and OA were observed at 1716 cm^−1^ and 1682 cm^−1^, respectively, while in the co-crystal, the former now appears at 1734 cm^−1^. Differential Scanning Calorimetric (DSC) analysis indicated the melting points of NAD and OA to be 478.9 K and 387.8 K, respectively, while the melting point of the co-crystal is 438.8 K.

## 6. Hirshfeld Surface analysis

**Figure 5:**
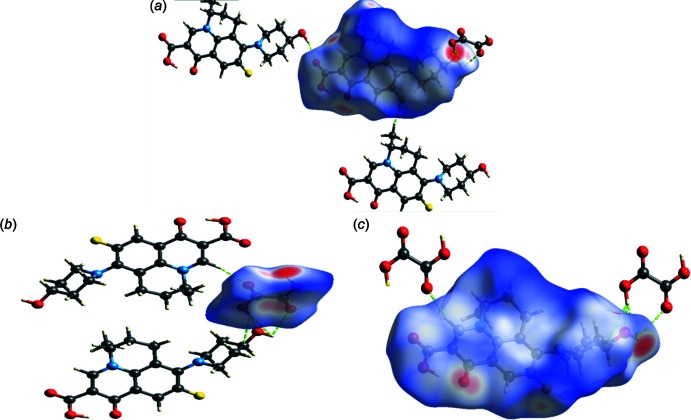
Calculated Hirshfeld surfaces mapped over *d*_norm_ for (*a*) NAD, (*b*) OA and (*c*) the NAD–OA co-crystal to visualize the inter­molecular inter­actions.

**Figure 6:**
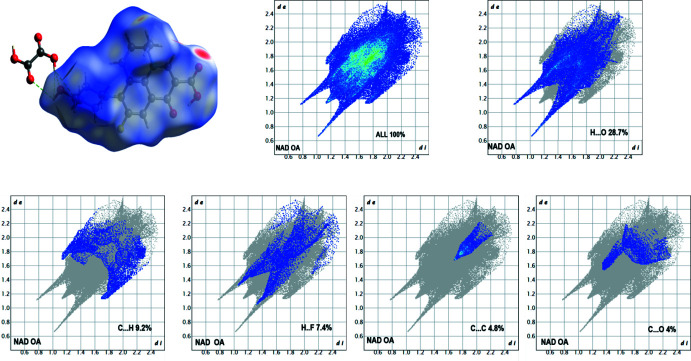
Two-dimensional fingerprint plots and relative contribution of various inter­actions to the Hirshfeld surface of the NAD mol­ecule.

**Figure 7:**
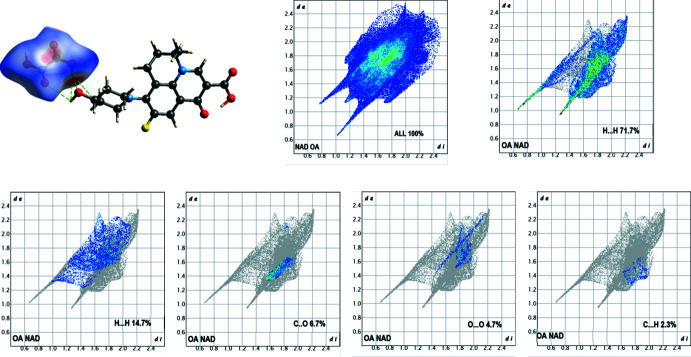
Two-dimensional fingerprint plots and relative contribution of various inter­actions to the Hirshfeld surface of the OA mol­ecule.

Hirshfeld analyses performed using *Crystal Explorer17* (Spackman *et al.*, 2009 ▸, 2021 ▸) are shown in Fig. 5 ▸. The surface mapping of this function highlights the donor and acceptor equally and it is therefore a powerful tool for analyzing inter­molecular inter­actions such as hydrogen bonds. Inter­molecular inter­actions within the crystal were mapped by *d*_norm_ and were determined for NAD and OA separately, as well as for the adduct of the two [Fig. 5 ▸(*a*)–(*c*), respectively (Bairagi *et al.*, 2019 ▸)]. The inter­actions generating the crystal packing were investigated from the Hirshfeld analysis using the two-dimensional fingerprint plots (Fig. 6 ▸). These show that for NAD, H⋯H contacts make the highest contribution to the inter­actions (43.4%), while O⋯H/H⋯O contribute 28.7%, C⋯H/H⋯C 9.2% and F⋯H/H—F 7.4%. The smallest contributions are from C⋯O and O⋯O contacts (4.8% and 0.7%, respectively). The two-dimensional fingerprint plots for oxalic acid (Fig. 7 ▸) show that O⋯H/H⋯O contacts make the highest contribution (71.7%), with H⋯H at 14.7%, and the smallest inter­actions being C⋯O (6.7%) and O⋯O (4.7%).

## 7. Refinement

Crystal data, data collection and structure refinement details are summarized in Table 2 ▸. C-bound H atoms were positioned geometrically (C—H = 0.93–0.98 Å) and refined as riding with *U*_iso_(H) = 1.2–1.5*U*_eq_(C). C-bound O atoms were freely refined.

## Supplementary Material

Crystal structure: contains datablock(s) I. DOI: 10.1107/S2056989023002244/mw2196sup1.cif

Structure factors: contains datablock(s) I. DOI: 10.1107/S2056989023002244/mw2196Isup2.hkl

Supporting information file. DOI: 10.1107/S2056989023002244/mw2196Isup3.cml

CCDC reference: 2194837

Additional supporting information:  crystallographic information; 3D view; checkCIF report

## Figures and Tables

**Table 1:** Hydrogen-bond geometry (Å, °)

*D*—H⋯*A*	*D*—H	H⋯*A*	*D*⋯*A*	*D*—H⋯*A*
C15—H15⋯O5^i^	0.93	2.35	3.266 (3)	167
C19—H19*B*⋯F1^ii^	0.96	2.50	3.456 (4)	174
O4—H4⋯O1^iii^	0.87 (4)	1.99 (4)	2.833 (3)	161 (4)
O2—H2⋯O3	0.87 (5)	1.68 (5)	2.536 (3)	165 (5)
O6—H6⋯O4	0.81 (4)	1.85 (4)	2.644 (3)	169 (4)

Symmetry codes: (i) 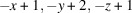 ; (ii) 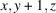 ; (iii) 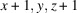 .

**Table 2:** Experimental details

Crystal data	
Chemical formula	C_19_H_21_FN_2_O_4_·0.5C_2_H_2_O_4_
*M*_r_	405.39
Crystal system, space group	Triclinic, *P* 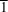
Temperature (K)	297
*a*, *b*, *c* (Å)	8.8187 (4), 9.6963 (4), 12.3804 (6)
α, β, γ (°)	100.099 (2), 97.556 (2), 109.858 (2)
*V* (Å^3^)	959.16 (8)
*Z*	2
Radiation type	Mo *K*α
μ (mm^−1^)	0.11
Crystal size (mm)	0.23 × 0.18 × 0.05

Data collection	
Diffractometer	Bruker D8 VENTURE with PHOTON II detector
Absorption correction	Multi-scan (*SADABS*; Bruker, 2016 ▸)
*T*_min_, *T*_max_	0.680, 0.960
No. of measured, independent and observed [*I* > 2σ(*I*)] reflections	40338, 3753, 2936
*R*_int_	0.066
(sin θ/λ)_max_ (Å^−1^)	0.617

Refinement	
*R*[*F* ^2^ > 2σ(*F* ^2^)], *wR*(*F* ^2^), *S*	0.062, 0.204, 1.07
No. of reflections	3753
No. of parameters	274
H-atom treatment	H atoms treated by a mixture of independent and constrained refinement
Δρ_max_, Δρ_min_ (e Å^−3^)	0.82, −0.37

Computer programs: *APEX3*, *SAINT* and *XPREP* (Bruker, 2016 ▸), *SHELXT2018/2* (Sheldrick, 2015*a* ▸), *SHELXL2018/3* (Sheldrick, 2015*b* ▸), *ORTEP-3 for Windows* (Farrugia, 2012 ▸) and *Mercury* (Macrae *et al.*, *et al.*, 2020 ▸).

## supplementary crystallographic information

### Crystal data

**Table:**

C_19_H_21_FN_2_O_4_·0.5C_2_H_2_O_4_	*Z* = 2
*M**_r_* = 405.39	*F*(000) = 426
Triclinic, *P*1	*D*_x_ = 1.404 Mg m^−^^3^
*a* = 8.8187 (4) Å	Mo *K*α radiation, λ = 0.71073 Å
*b* = 9.6963 (4) Å	Cell parameters from 9915 reflections
*c* = 12.3804 (6) Å	θ = 3.2–30.5°
α = 100.099 (2)°	µ = 0.11 mm^−^^1^
β = 97.556 (2)°	*T* = 297 K
γ = 109.858 (2)°	Block, colourless
*V* = 959.16 (8) Å^3^	0.23 × 0.18 × 0.05 mm

### Data collection

**Table:**

Bruker D8 VENTURE diffractometer with PHOTON II detector	3753 independent reflections
Radiation source: fine-focus sealed tube	2936 reflections with *I* > 2σ(*I*)
Graphite monochromator	*R*_int_ = 0.066
ω and φ scan	θ_max_ = 26.0°, θ_min_ = 2.5°
Absorption correction: multi-scan (SADABS; Bruker, 2016)	*h* = −10→10
*T*_min_ = 0.680, *T*_max_ = 0.960	*k* = −11→11
40338 measured reflections	*l* = −15→15

### Refinement

**Table:**

Refinement on *F*^2^	Primary atom site location: dual
Least-squares matrix: full	Secondary atom site location: difference Fourier map
*R*[*F*^2^ > 2σ(*F*^2^)] = 0.062	Hydrogen site location: mixed
*wR*(*F*^2^) = 0.204	H atoms treated by a mixture of independent and constrained refinement
*S* = 1.07	*w* = 1/[σ^2^(*F*_o_^2^) + (0.1129*P*)^2^ + 0.4888*P*] where *P* = (*F*_o_^2^ + 2*F*_c_^2^)/3
3753 reflections	(Δ/σ)_max_ < 0.001
274 parameters	Δρ_max_ = 0.82 e Å^−^^3^
0 restraints	Δρ_min_ = −0.36 e Å^−^^3^

### Special details

**Table:**

Geometry. All esds (except the esd in the dihedral angle between two l.s. planes) are estimated using the full covariance matrix. The cell esds are taken into account individually in the estimation of esds in distances, angles and torsion angles; correlations between esds in cell parameters are only used when they are defined by crystal symmetry. An approximate (isotropic) treatment of cell esds is used for estimating esds involving l.s. planes.

### Fractional atomic coordinates and isotropic or equivalent isotropic displacement parameters (Å^2^)

**Table:**

	*x*	*y*	*z*	*U*_iso_*/*U*_eq_	
C1	0.7236 (3)	0.5092 (3)	0.5094 (2)	0.0446 (6)	
H1A	0.786613	0.448224	0.487203	0.054*	
H1B	0.610683	0.442369	0.503059	0.054*	
C2	0.7960 (3)	0.6000 (3)	0.6298 (2)	0.0483 (6)	
H2A	0.729953	0.657665	0.652556	0.058*	
H2B	0.793775	0.531942	0.678930	0.058*	
C3	0.9705 (3)	0.7053 (3)	0.64044 (19)	0.0409 (5)	
H3	1.036372	0.644849	0.620463	0.049*	
C4	0.9767 (4)	0.8070 (3)	0.5603 (2)	0.0622 (8)	
H4A	1.090432	0.869057	0.563484	0.075*	
H4B	0.919365	0.873448	0.582464	0.075*	
C5	0.8971 (4)	0.7143 (3)	0.4413 (2)	0.0553 (7)	
H5A	0.895998	0.781043	0.391546	0.066*	
H5B	0.960975	0.655339	0.416318	0.066*	
C6	0.6212 (3)	0.5592 (3)	0.32960 (19)	0.0373 (5)	
C7	0.5750 (3)	0.4119 (3)	0.2639 (2)	0.0389 (5)	
C8	0.4657 (3)	0.3567 (3)	0.16488 (19)	0.0383 (5)	
H8	0.437499	0.257443	0.125721	0.046*	
C9	0.3953 (3)	0.4508 (2)	0.12182 (18)	0.0341 (5)	
C10	0.4407 (3)	0.5999 (3)	0.18365 (19)	0.0363 (5)	
C11	0.5518 (3)	0.6541 (3)	0.2878 (2)	0.0438 (6)	
C12	0.5871 (5)	0.8099 (3)	0.3573 (3)	0.0769 (11)	
H12A	0.686634	0.881068	0.343893	0.092*	
H12B	0.603838	0.809967	0.436303	0.092*	
C13	0.4368 (6)	0.8586 (4)	0.3244 (3)	0.0826 (11)	
H13A	0.338644	0.791540	0.342407	0.099*	
H13B	0.460161	0.960507	0.366295	0.099*	
C14	0.4095 (4)	0.8514 (3)	0.2020 (2)	0.0561 (7)	
H14	0.311979	0.876237	0.183147	0.067*	
C15	0.2626 (3)	0.6402 (3)	0.0415 (2)	0.0434 (6)	
H15	0.218651	0.705629	0.015085	0.052*	
C16	0.2152 (3)	0.4969 (3)	−0.02349 (19)	0.0394 (5)	
C17	0.2785 (3)	0.3925 (3)	0.01480 (19)	0.0368 (5)	
C18	0.0973 (3)	0.4525 (3)	−0.1316 (2)	0.0477 (6)	
C19	0.5417 (6)	0.9504 (4)	0.1580 (4)	0.0956 (13)	
H19A	0.507225	0.933707	0.078356	0.143*	
H19B	0.564730	1.053863	0.192970	0.143*	
H19C	0.639225	0.928141	0.173803	0.143*	
N1	0.3677 (3)	0.6916 (2)	0.14029 (17)	0.0433 (5)	
N2	0.7286 (3)	0.6134 (2)	0.43631 (16)	0.0421 (5)	
O1	0.0470 (3)	0.5392 (3)	−0.17064 (18)	0.0693 (6)	
O2	0.0451 (3)	0.3099 (2)	−0.18472 (17)	0.0653 (6)	
O3	0.2365 (3)	0.2583 (2)	−0.03941 (15)	0.0532 (5)	
O4	1.0425 (3)	0.7963 (2)	0.75214 (15)	0.0552 (5)	
F1	0.6444 (2)	0.31952 (17)	0.30200 (13)	0.0590 (5)	
H4	1.051 (5)	0.732 (5)	0.791 (3)	0.093 (13)*	
H2	0.103 (6)	0.276 (5)	−0.142 (4)	0.112 (16)*	
C20	0.9337 (3)	1.0187 (3)	0.9660 (2)	0.0412 (6)	
O5	0.8664 (3)	1.0949 (2)	1.01002 (15)	0.0561 (5)	
O6	0.8992 (3)	0.9631 (2)	0.85737 (15)	0.0536 (5)	
H6	0.950 (5)	0.913 (5)	0.834 (4)	0.092 (14)*	

### Atomic displacement parameters (Å^2^)

**Table:**

	*U*^11^	*U*^22^	*U*^33^	*U*^12^	*U*^13^	*U*^23^
C1	0.0415 (13)	0.0485 (13)	0.0435 (14)	0.0148 (11)	0.0034 (10)	0.0178 (11)
C2	0.0440 (14)	0.0684 (17)	0.0382 (13)	0.0241 (12)	0.0092 (10)	0.0195 (12)
C3	0.0438 (13)	0.0481 (13)	0.0298 (11)	0.0211 (11)	0.0023 (9)	0.0026 (9)
C4	0.0716 (19)	0.0521 (16)	0.0402 (15)	0.0018 (14)	−0.0051 (13)	0.0095 (12)
C5	0.0579 (17)	0.0545 (15)	0.0343 (13)	0.0001 (13)	−0.0008 (11)	0.0119 (11)
C6	0.0375 (12)	0.0398 (12)	0.0336 (12)	0.0151 (10)	0.0024 (9)	0.0087 (9)
C7	0.0409 (13)	0.0393 (12)	0.0414 (13)	0.0216 (10)	0.0055 (10)	0.0108 (10)
C8	0.0426 (13)	0.0344 (11)	0.0389 (12)	0.0181 (10)	0.0067 (10)	0.0048 (9)
C9	0.0355 (11)	0.0354 (11)	0.0334 (11)	0.0143 (9)	0.0094 (9)	0.0089 (9)
C10	0.0409 (12)	0.0362 (11)	0.0350 (12)	0.0180 (9)	0.0061 (9)	0.0099 (9)
C11	0.0535 (15)	0.0369 (12)	0.0385 (13)	0.0183 (11)	0.0005 (11)	0.0063 (10)
C12	0.121 (3)	0.0481 (16)	0.0536 (18)	0.0454 (18)	−0.0242 (18)	−0.0043 (13)
C13	0.114 (3)	0.065 (2)	0.067 (2)	0.051 (2)	−0.004 (2)	−0.0057 (16)
C14	0.0712 (19)	0.0397 (13)	0.0593 (17)	0.0322 (13)	−0.0038 (14)	0.0053 (12)
C15	0.0500 (14)	0.0463 (13)	0.0405 (13)	0.0241 (11)	0.0058 (11)	0.0166 (10)
C16	0.0411 (13)	0.0466 (13)	0.0350 (12)	0.0179 (10)	0.0100 (10)	0.0161 (10)
C17	0.0378 (12)	0.0392 (12)	0.0337 (11)	0.0140 (9)	0.0091 (9)	0.0090 (9)
C18	0.0507 (15)	0.0589 (15)	0.0347 (13)	0.0191 (12)	0.0081 (11)	0.0170 (11)
C19	0.114 (3)	0.0472 (18)	0.110 (3)	0.0189 (19)	0.006 (3)	0.0145 (19)
N1	0.0534 (12)	0.0386 (11)	0.0415 (11)	0.0242 (9)	0.0019 (9)	0.0099 (8)
N2	0.0453 (11)	0.0430 (11)	0.0349 (10)	0.0148 (9)	0.0002 (8)	0.0107 (8)
O1	0.0829 (16)	0.0725 (14)	0.0525 (12)	0.0332 (12)	−0.0100 (11)	0.0234 (10)
O2	0.0846 (16)	0.0617 (13)	0.0379 (11)	0.0229 (11)	−0.0088 (10)	0.0073 (9)
O3	0.0690 (13)	0.0427 (10)	0.0407 (10)	0.0210 (9)	−0.0027 (8)	0.0015 (7)
O4	0.0696 (13)	0.0608 (12)	0.0309 (9)	0.0292 (10)	−0.0026 (8)	−0.0004 (8)
F1	0.0711 (11)	0.0514 (9)	0.0583 (10)	0.0405 (8)	−0.0105 (8)	0.0040 (7)
C20	0.0503 (14)	0.0362 (11)	0.0362 (12)	0.0201 (10)	−0.0004 (10)	0.0053 (9)
O5	0.0732 (13)	0.0612 (12)	0.0443 (10)	0.0454 (10)	0.0012 (9)	0.0052 (8)
O6	0.0681 (13)	0.0586 (11)	0.0337 (9)	0.0343 (10)	−0.0064 (8)	0.0006 (8)

### Geometric parameters (Å, º)

**Table:**

C1—N2	1.464 (3)	C11—C12	1.508 (4)
C1—C2	1.520 (4)	C12—C13	1.580 (5)
C1—H1A	0.9700	C12—H12A	0.9700
C1—H1B	0.9700	C12—H12B	0.9700
C2—C3	1.504 (4)	C13—C14	1.488 (5)
C2—H2A	0.9700	C13—H13A	0.9700
C2—H2B	0.9700	C13—H13B	0.9700
C3—O4	1.433 (3)	C14—C19	1.473 (6)
C3—C4	1.510 (4)	C14—N1	1.497 (3)
C3—H3	0.9800	C14—H14	0.9800
C4—C5	1.519 (4)	C15—N1	1.333 (3)
C4—H4A	0.9700	C15—C16	1.371 (3)
C4—H4B	0.9700	C15—H15	0.9300
C5—N2	1.461 (3)	C16—C17	1.429 (3)
C5—H5A	0.9700	C16—C18	1.478 (3)
C5—H5B	0.9700	C17—O3	1.258 (3)
C6—C11	1.399 (3)	C18—O1	1.216 (3)
C6—C7	1.406 (3)	C18—O2	1.313 (3)
C6—N2	1.417 (3)	C19—H19A	0.9600
C7—C8	1.351 (3)	C19—H19B	0.9600
C7—F1	1.358 (3)	C19—H19C	0.9600
C8—C9	1.404 (3)	O2—H2	0.87 (5)
C8—H8	0.9300	O4—H4	0.87 (4)
C9—C10	1.405 (3)	C20—O5	1.199 (3)
C9—C17	1.456 (3)	C20—O6	1.311 (3)
C10—N1	1.400 (3)	C20—C20^i^	1.531 (5)
C10—C11	1.407 (3)	O6—H6	0.81 (4)
			
N2—C1—C2	108.9 (2)	C11—C12—C13	109.2 (3)
N2—C1—H1A	109.9	C11—C12—H12A	109.8
C2—C1—H1A	109.9	C13—C12—H12A	109.8
N2—C1—H1B	109.9	C11—C12—H12B	109.8
C2—C1—H1B	109.9	C13—C12—H12B	109.8
H1A—C1—H1B	108.3	H12A—C12—H12B	108.3
C3—C2—C1	110.4 (2)	C14—C13—C12	108.7 (3)
C3—C2—H2A	109.6	C14—C13—H13A	110.0
C1—C2—H2A	109.6	C12—C13—H13A	110.0
C3—C2—H2B	109.6	C14—C13—H13B	110.0
C1—C2—H2B	109.6	C12—C13—H13B	110.0
H2A—C2—H2B	108.1	H13A—C13—H13B	108.3
O4—C3—C2	112.4 (2)	C19—C14—C13	118.6 (3)
O4—C3—C4	109.1 (2)	C19—C14—N1	108.4 (3)
C2—C3—C4	110.1 (2)	C13—C14—N1	108.4 (2)
O4—C3—H3	108.4	C19—C14—H14	107.0
C2—C3—H3	108.4	C13—C14—H14	107.0
C4—C3—H3	108.4	N1—C14—H14	107.0
C3—C4—C5	110.6 (2)	N1—C15—C16	124.0 (2)
C3—C4—H4A	109.5	N1—C15—H15	118.0
C5—C4—H4A	109.5	C16—C15—H15	118.0
C3—C4—H4B	109.5	C15—C16—C17	119.7 (2)
C5—C4—H4B	109.5	C15—C16—C18	119.1 (2)
H4A—C4—H4B	108.1	C17—C16—C18	121.2 (2)
N2—C5—C4	110.3 (2)	O3—C17—C16	122.8 (2)
N2—C5—H5A	109.6	O3—C17—C9	121.5 (2)
C4—C5—H5A	109.6	C16—C17—C9	115.8 (2)
N2—C5—H5B	109.6	O1—C18—O2	120.3 (2)
C4—C5—H5B	109.6	O1—C18—C16	123.6 (3)
H5A—C5—H5B	108.1	O2—C18—C16	116.1 (2)
C11—C6—C7	117.4 (2)	C14—C19—H19A	109.5
C11—C6—N2	119.0 (2)	C14—C19—H19B	109.5
C7—C6—N2	123.6 (2)	H19A—C19—H19B	109.5
C8—C7—F1	118.0 (2)	C14—C19—H19C	109.5
C8—C7—C6	123.9 (2)	H19A—C19—H19C	109.5
F1—C7—C6	118.1 (2)	H19B—C19—H19C	109.5
C7—C8—C9	119.3 (2)	C15—N1—C10	120.8 (2)
C7—C8—H8	120.4	C15—N1—C14	118.32 (19)
C9—C8—H8	120.4	C10—N1—C14	120.8 (2)
C8—C9—C10	118.7 (2)	C6—N2—C5	116.53 (19)
C8—C9—C17	119.6 (2)	C6—N2—C1	118.84 (19)
C10—C9—C17	121.7 (2)	C5—N2—C1	111.8 (2)
N1—C10—C9	118.0 (2)	C18—O2—H2	101 (3)
N1—C10—C11	120.8 (2)	C3—O4—H4	104 (3)
C9—C10—C11	121.1 (2)	O5—C20—O6	122.4 (2)
C6—C11—C10	119.5 (2)	O5—C20—C20^i^	121.6 (3)
C6—C11—C12	120.2 (2)	O6—C20—C20^i^	116.1 (3)
C10—C11—C12	120.1 (2)	C20—O6—H6	117 (3)
			
N2—C1—C2—C3	−58.9 (3)	N1—C15—C16—C18	179.9 (2)
C1—C2—C3—O4	178.4 (2)	C15—C16—C17—O3	178.0 (2)
C1—C2—C3—C4	56.6 (3)	C18—C16—C17—O3	−1.1 (4)
O4—C3—C4—C5	−178.5 (2)	C15—C16—C17—C9	−1.7 (3)
C2—C3—C4—C5	−54.7 (3)	C18—C16—C17—C9	179.2 (2)
C3—C4—C5—N2	55.8 (4)	C8—C9—C17—O3	1.3 (3)
C11—C6—C7—C8	−1.5 (4)	C10—C9—C17—O3	−178.9 (2)
N2—C6—C7—C8	176.2 (2)	C8—C9—C17—C16	−178.9 (2)
C11—C6—C7—F1	178.4 (2)	C10—C9—C17—C16	0.8 (3)
N2—C6—C7—F1	−3.8 (4)	C15—C16—C18—O1	5.1 (4)
F1—C7—C8—C9	−178.3 (2)	C17—C16—C18—O1	−175.8 (2)
C6—C7—C8—C9	1.6 (4)	C15—C16—C18—O2	−173.8 (2)
C7—C8—C9—C10	−0.2 (3)	C17—C16—C18—O2	5.3 (4)
C7—C8—C9—C17	179.5 (2)	C16—C15—N1—C10	1.3 (4)
C8—C9—C10—N1	−179.2 (2)	C16—C15—N1—C14	179.5 (3)
C17—C9—C10—N1	1.1 (3)	C9—C10—N1—C15	−2.2 (4)
C8—C9—C10—C11	−1.3 (3)	C11—C10—N1—C15	179.9 (2)
C17—C9—C10—C11	179.0 (2)	C9—C10—N1—C14	179.6 (2)
C7—C6—C11—C10	0.0 (4)	C11—C10—N1—C14	1.7 (4)
N2—C6—C11—C10	−177.9 (2)	C19—C14—N1—C15	−83.7 (3)
C7—C6—C11—C12	175.9 (3)	C13—C14—N1—C15	146.4 (3)
N2—C6—C11—C12	−1.9 (4)	C19—C14—N1—C10	94.5 (3)
N1—C10—C11—C6	179.2 (2)	C13—C14—N1—C10	−35.4 (4)
C9—C10—C11—C6	1.4 (4)	C11—C6—N2—C5	−78.3 (3)
N1—C10—C11—C12	3.3 (4)	C7—C6—N2—C5	104.0 (3)
C9—C10—C11—C12	−174.6 (3)	C11—C6—N2—C1	143.2 (2)
C6—C11—C12—C13	−151.7 (3)	C7—C6—N2—C1	−34.5 (3)
C10—C11—C12—C13	24.2 (4)	C4—C5—N2—C6	159.0 (2)
C11—C12—C13—C14	−57.1 (4)	C4—C5—N2—C1	−59.7 (3)
C12—C13—C14—C19	−62.3 (4)	C2—C1—N2—C6	−158.9 (2)
C12—C13—C14—N1	61.7 (4)	C2—C1—N2—C5	60.9 (3)
N1—C15—C16—C17	0.7 (4)		

Symmetry code: (i) −*x*+2, −*y*+2, −*z*+2.

### Hydrogen-bond geometry (Å, º)

**Table:**

*D*—H···*A*	*D*—H	H···*A*	*D*···*A*	*D*—H···*A*
C15—H15···O5^ii^	0.93	2.35	3.266 (3)	167
C19—H19*B*···F1^iii^	0.96	2.50	3.456 (4)	174
O4—H4···O1^iv^	0.87 (4)	1.99 (4)	2.833 (3)	161 (4)
O2—H2···O3	0.87 (5)	1.68 (5)	2.536 (3)	165 (5)
O6—H6···O4	0.81 (4)	1.85 (4)	2.644 (3)	169 (4)

Symmetry codes: (ii) −*x*+1, −*y*+2, −*z*+1; (iii) *x*, *y*+1, *z*; (iv) *x*+1, *y*, *z*+1.
